# A simplified approach to true molar intrusion

**DOI:** 10.1186/1746-160X-8-30

**Published:** 2012-11-07

**Authors:** Stefanie Flieger, Thomas Ziebura, Johannes Kleinheinz, Dirk Wiechmann

**Affiliations:** 1 Department of Orthodontics, University of Münster, Albert-Schweitzer-Campus 1, 48149, Münster, Germany; 2 Department of Maxillofacial Surgery, University of Münster, Albert-Schweitzer-Campus 1, 48149, Münster, Germany; 3 Department of Orthodontics, Medizinische Hochschule Hannover, Carl-Neuberg-Str. 1, 30625, Hannover, Germany

## Abstract

**Background:**

Orthodontic management of anterior open bites is a demanding task for orthodontists. Molar intrusion as a primary means of open bite correction entails the need for appropriate anchorage. Orthodontic mini implants can provide the required mechanical support. The suggested procedure aims to reduce the risk of complications such as root damage or soft tissue irritations while minimizing overall complexity.

**Methods:**

Three female patients aged 14, 18 and 19 years who decided against a surgical correction were treated with a device consisting of mini implants in the palatal slope, a palatal bar and intrusion cantilevers.

**Results:**

In all three patients, an open bite reduction of more than a millimeter occurred within four months. An anterior overbite of 2 mm or more could be established within 6 to 9 months.

**Conclusions:**

The method presented in this article enables the practitioner to use mini implants in an easily accessible insertion site. A lab-side procedure is optional but not required.

## Background

The management of anterior open bites is considered one of the most difficult tasks in orthodontics.

In growing patients, functional or orthopedic approaches can be applicable [1-3]. Without growth, only orthognathic surgery and orthodontic tooth movement remain as means of therapeutic intervention.

Orthodontic solutions may involve an unfavorable extrusion of the incisors and may result in an increased display of gingiva [4]. In many cases, posterior intrusion is favorable but it requires appropriate anchorage. A high-pull headgear can provide for an adequately directed force but depends on patient cooperation. Furthermore, it is usually not an option in adults. Skeletal anchorage using mini plates has been proven efficient in the management of open bites [5]. However, this approach requires incision and suture.

Various mini implant insertion sites are available for the purpose of molar intrusion. Interradicular insertion bears the risk of root damage. Even after correct implant insertion, root contact may still occur later due to the progress of the intrusion, which may impair implant stability. Insertion beyond the attached gingiva may also lead to an increased failure rate [6]. The palate as a skeletal anchorage site has been described by several authors and for different treatment tasks [7,8]. It offers good bone support [9-11].

The aim of this work is to present an approach that does not require incision, interradicular implant placement or placement in the movable alveolar mucosa while keeping the complexity of the orthodontic mechanics at a minimum.

## Method

### Implant and insertion

**Figure 1:**
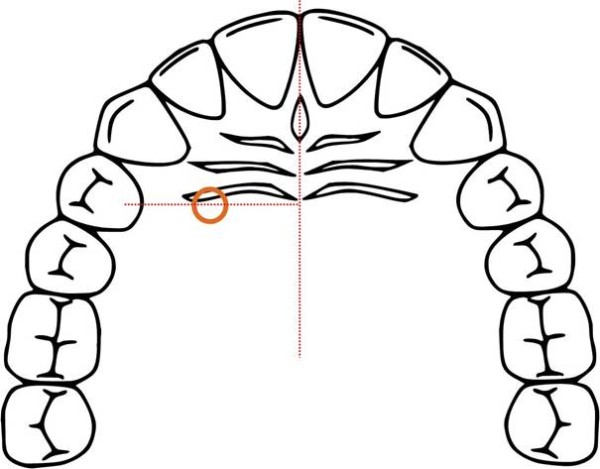
Insertion site location.

Instead of a gingival collar the Jet Screw (JS) type mini implants (Promedia Medizintechnik GmbH, Siegen, Germany) used in this work have a long neck which widens towards the implant head. This makes them applicable in areas covered by thick soft tissue. They are advertised for use with the TopJet Distalizer (H. Winsauer, Bregenz, Austria; Promedia Medizintechnik GmbH, Siegen, Germany). The insertion site recommended by the manufacturer is located at half of the distance of the perpendicular line segment from the raphe to the palatal cusp tip of the first bicuspid (Figure 1).

The mini implants were inserted according to the following protocol:

•Informed consent regarding potential risks, complications and behavior via standardized documents

•surface anesthesia with 1% lidocaine spray applied using a cotton ball

•infiltration using 4% articaine solution

•mouth rinse with 0.2% chlorhexidine digluconate

•assessment of gingival thickness using a probe

•choice of JS neck length: 3 mm neck for gingival thicknesses not exceeding 3.5 mm, otherwise 5 mm

•insertion using a surgical handpiece or a hand screw driver

### Intrusion mechanics

**Figure 2:**
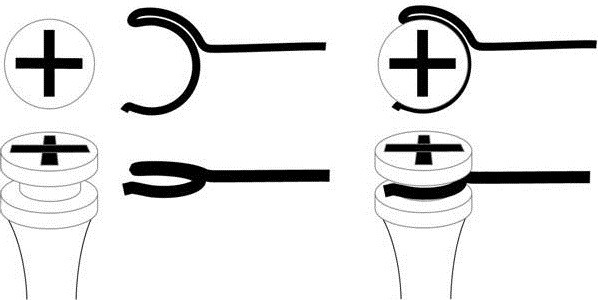
Connecting clip.

**Figure 3:**
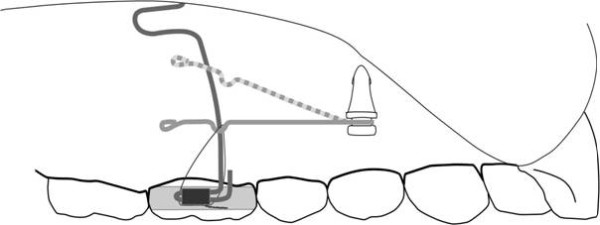
Intrusion mechanics – dotted line depicts location of intrusion cantilever before activation with steel ligature.

Posterior intrusion was achieved through distally extended cantilevers fabricated out of 16×22 stainless steel wire. The connection between implant neck and wire was established by bending the anterior end of the wire into the shape of a clip (Figure 2). This clip permitted for a rigid connection between the wire and the implant neck. The distal end of the wire had to be designed in a way that involves as little soft tissue irritation as possible. Therefore, a smooth hook shape was chosen. A gable bend distal of the clip caused the cantilever to point towards the roof of the palate (Figure 3).

A palatal bar served to avoid palatal tipping of the molars during intrusion. Also, it helped in preventing the cantilevers from interfering with the gingiva. Notably, the palatal bar was adjusted to leave at least 3 mm of space to the roof of the palate in order to obviate excessive soft tissue contact as the intrusion progresses.

Once attached to the mini implant, the cantilevers rest on the palatal bar. To establish the desired intrusive force level, they were then tied to the molar bands with steel ligatures. An initial force level of 60 cN per side was chosen for the intrusion of the first molars and verified using a spring balance. A force of 200 cN per side was applied when second bicuspids, first molars and second molars were being intruded simultaneously. During subsequent appointments the ligatures were gradually tightened to maintain the intrusive force.

### Patient 1

**Figure 4:**
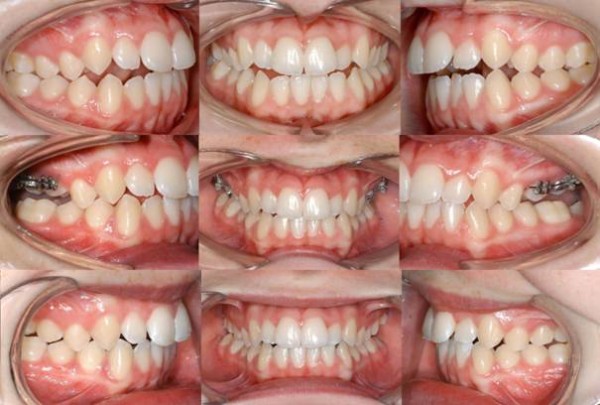
Treatment stages for case 1: initial situation (upper row), over-correction (middle row) and four months after intrusion appliance removal (lower row).

An 18 year old woman with an anterior open bite was treated according to above method (Figure 4). The negative overbite amounted to 3 mm between the upper and lower right lateral incisors. The smallest value - 0 mm - was established between the upper and lower left central incisors. The cephalometric analysis revealed a clockwise rotation of the mandible as well as a regular palatal plane angle.

**Figure 5:**
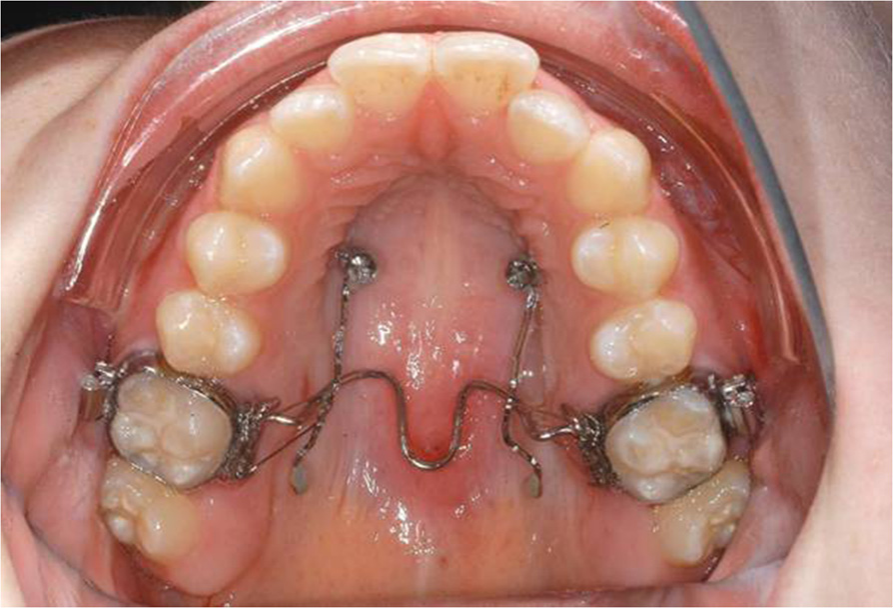
Intrusion mechanics for case 1.

Initially, only the upper first molars were intruded (Figure 5). After five months of treatment, when a vertical distance between upper and lower first molars became visible, brackets were bonded to the maxillary second bicuspids and second molars and short wire segments (.012” NiTi) were inserted. The wire segments were subsequently replaced by .016”×.022” NiTi and .016”×.022” stainless steel wires.

**Figure 6:**
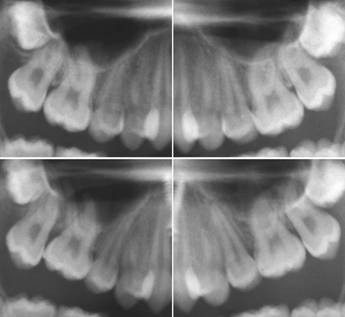
Case 1: apical situation before (upper row) and after treatment (lower row).

After removal of the intrusion cantilevers three months later, treatment ceased for two months in order to estimate the amount of relapse. Panoramic x-ray images served to assess the apical situation and to identify possible root resorptions (Figure 6).

**Figure 7:**
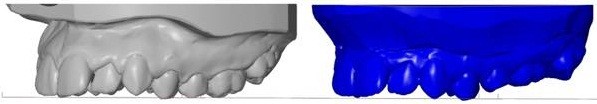
Case 1: aligned 3D scans of pre- and post-intrusion plaster casts.

**Figure 8:**
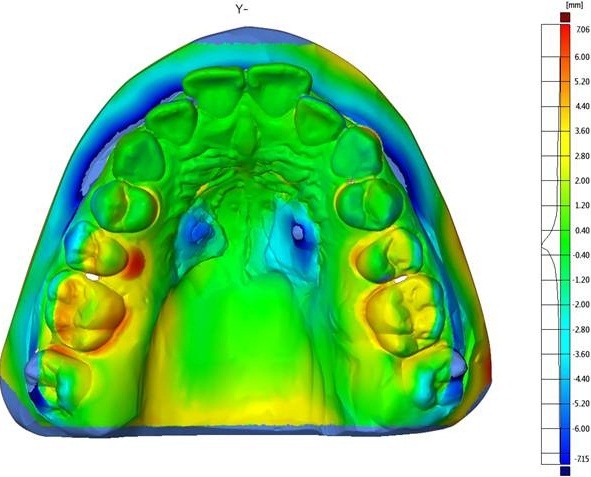
Case 1: displacement map to visualize the amount of intrusion.

The amount of dentoalveolar change was assessed clinically as well as by means of digitized plaster casts. An optical 3D-scanner and a spezialzed software (GOM mbH, Braunschweig, Germany) served to obtain the according data and to overlay initial and post-intrusion maxillary casts using the best fit method (Figure 7). The anterior hard palate and the untreated incisors provided a reference for this procedure. A displacement map was generated to visualize the changes (Figure 8).

### Patient 2

**Figure 9:**
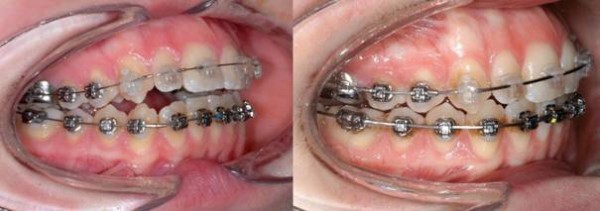
Case 2 before and after molar intrusion.

In a 19 year old woman, the intrusion procedure was commenced when a fixed appliance was already in situ and the leveling and aligning stages of treatment were almost complete (Figure 9). She exhibited an anterior open bite of 2.5 mm, a class I molar relationship on the left side and a quarter of a unit class II on the right. No intermaxillary elastics were used.

### Patient 3

**Figure 10:**
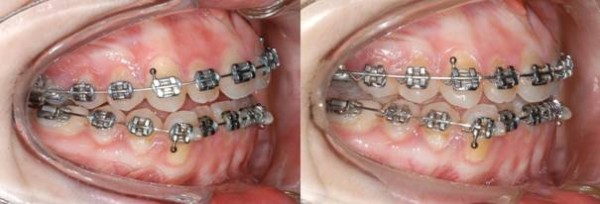
Case 3 before and after molar intrusion.

A 14 year old girl received a similar treatment Figure 10. The anterior open bite amounted to 2 mm after leveling. Just as in the other cases, treatment was conducted without intermaxillary elastics.

## Results

### Patient 1

At the time of the most extensive over-correction, the anterior open bite was remediated and an iatrogenic lateral open bite of 3.5 mm on the right side and 4 mm on the left had occurred. In the following four months, the lateral open bite closed incompletely. A lateral open bite of 1.5 mm on each side remained. The anterior overbite persisted. It amounted to 1 mm at the right lateral incisors and to 4 mm in the left central incisor area.

The x-ray images (Figure 6) showed no discernable sign of root resorption although a change of the first molars’ position in relation to the maxillary sinus floor became visible.

In Figure 7, the digitized models are shown in conjunction with a reference plane.

In addition to the vertical correction, an improvement of the molar relationship was observed. On the right side, a class I molar relationship was established. On the left side, a quarter of a unit class II remained.

The transversal distance between the upper first molars increased by 2.5 mm which may be attributed to a slight over-activation of the palatal bar.

### Patient 2

Within five months, the anterior open bite was closed and a lateral open bite of 1 mm was established. Occlusal contacts remained between the second molars and first bicuspids. On both sides, a super class I occlusion resulted.

**Figure 11:**
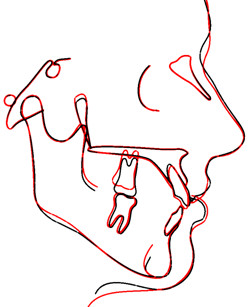
Superimposition of cephalometric tracings on the Ba-N line for case 2.

**Figure 12:**
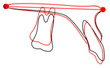
Superimposition on the palatal plane for case 2.

The cephalometric tracings (Figure 11 and Figure 12) reveal a molar intrusion of 4 mm. Notably, they also show a slight extrusion of the maxillary incisors.

### Patient 3

Five months of intrusion resulted in an anterior overbite and a vertical distance of 2 mm between upper and lower first molars.

## Discussion

The aspired treatment objective was reached in all cases. The clinically visible open bite reduction can be attributed to the intrusion mechanics since no intermaxillary elastics were used. Still, an incisor extrusion was observed in case 2. It may result from the fact that the intrusive force was applied distal to the center of resistance of the maxillary dentition entailing a clockwise rotation of the entire dental arch.

In terms of implant placement, the suggested procedure benefits from an insertion site which is accessible with a surgical hand piece as well as with a straight hand screw driver unless pathological limitations of mouth opening are present. Slight deviations regarding the implants’ position as well as their angulation can be compensated for by adapting the cantilevers accordingly.

Maxillary molar intrusion may also be conducted by using miniplates instead of mini implants [12]. However, this approach involves an incision and a more complex surgical procedure in both insertion and removal of the anchorage unit.

Mini implant insertion in the buccal alveolus may bear the risk of root damage. Additionally, in order to achieve a favorable force vector, a high insertion location in the area of the movable mucosa would be necessary, which is suboptimal in terms of implant survival [6]. The palatal alveolus is another known insertion site for molar intrusion, too [12]. It eliminates the issues linked to the movable mucosa but still requires interradicular implant placement. While successful molar intrusion has been described using single screws in the posterior palate, this insertion location is less easily accessible and eliminates the option of using a hand screw driver [13]. Also, only the midline of the posterior palate offers sufficient bony support, raising the requirements on surgical accuracy [14].

The suggested procedure can be performed chair side. The clip connectors can be pre-fabricated. A lab-side fabrication of the palatal bar is optional.

Although the reliability of the presented method requires further investigation, the results appear propitious. The first case is especially insightful since the observed effect can be fully attributed to the intrusion mechanics. No brackets, elastics or other appliances were being used. It can, however, be argued that treatment might have been more efficient if molar intrusion had been performed simultaneously with leveling and aligning.

The intrusion cantilevers were fabricated out of .016”×.022” stainless steel wire. The decision for this material was made because TMA wires are more prone to breakage whereas NiTi wires cannot as easily be bent. A hybrid construction with a steel clip on one end and a NiTi lever is conceivable but was not deemed necessary. The sheer length of the levers provided for a sufficient level of elasticity. Finally, with regard to the possibility of root resorption, dissipating forces may even be favorable [15].

## Conclusion

While additional research is required, present results indicate that the proposed method is suitable for treatment of anterior open bites. It is advantageous in terms of surgical difficulty and mechanical complexity.

## Ethical approval

Written informed consent was obtained from all patients for publication of this report and the accompanying images. In all cases, a medical indication for the respective treatment was present. The surgical procedure constitutes a routine treatment. The authors declare that no ethical approval was necessary.

## Competing interests

The authors declare that they have no competing interests.

## Authors’ contributions

TZ suggested the original idea for the paper. TZ, SF and JK wrote the main part of the manuscript. JK and DW reviewed the paper for content, and reviewed and contributed to the writing of all iterations of the paper, including the final version of the manuscript. All authors read and approved the final manuscript.
